# Atypical Presentation of Undiagnosed Crouzon′s Syndrome With Pansinusitis, Orbital Cellulitis, and Altered Mentation Successfully Managed With Functional Endoscopic Sinus Surgery: A Case Report

**DOI:** 10.1155/crcc/8887878

**Published:** 2026-09-12

**Authors:** Parul Issar, Shivam Galav, Raunaq Goel, Ashesh Bhushan

**Affiliations:** ^1^ Department of Critical Care Medicine, Yatharth Super-Speciality Hospital, Greater Noida, India; ^2^ Department of Radio-Diagnosis, Yatharth Super-Speciality Hospital, Greater Noida, India; ^3^ Department of Ear, Nose and Throat Surgery, Yatharth Super-Speciality Hospital, Greater Noida, India

**Keywords:** atypical presentation, case report, craniosynostosis, Crouzon′s syndrome, FESS, meningitis, orbital cellulitis, pansinusitis

## Abstract

**Background:**

Crouzon′s syndrome is a rare autosomal dominant craniosynostosis disorder characterized by midfacial hypoplasia due to premature fusion of the coronal and sagittal sutures. Facial features are typical in the form of a hypoplastic maxilla, shallow orbits, and mandibular prognathism. The condition is usually diagnosed during infancy or early childhood because of characteristic craniofacial features. Delayed diagnosis during adolescence is uncommon, and severe infective complications as the initial presentation are exceedingly rare.

**Case Summary:**

A 14‐year‐old boy with no known craniofacial diagnosis presented to the emergency department with fever, headache, altered mentation, and left eye swelling. Examination revealed proptosis, chemosis, and purulent discharge from the left eye, along with midfacial hypoplasia and shallow orbits. Computed tomography and magnetic resonance imaging demonstrated extensive pansinusitis, orbital cellulitis, frontal bone osteomyelitis, and craniosynostosis‐related craniofacial abnormalities. Initial empirical antimicrobial therapy was started and subsequently escalated following the isolation of carbapenem‐resistant *Pseudomonas aeruginosa*. Owing to progressive disease, urgent functional endoscopic sinus surgery (FESS) was performed. The patient demonstrated rapid clinical improvement with resolution of orbital swelling and recovery of neurological status. Subsequent genetic testing confirmed an FGFR2 mutation, establishing the diagnosis of Crouzon syndrome.

**Conclusion:**

This case highlights a rare late presentation of previously undiagnosed Crouzon syndrome with life‐threatening infective complications. Recognition of craniofacial dysmorphism in patients presenting with severe sinonasal infections is essential for timely diagnosis and multidisciplinary management. Culture‐directed antimicrobial therapy combined with appropriately planned FESS can result in excellent clinical outcomes.

## 1. Introduction

CS is a rare autosomal dominant disorder with a characteristic craniofacial malformation. It was first described by Louis Edouard Octave Crouzon in 1912 as a syndrome of midfacial hypoplasia and shallow orbits due to premature synostosis of the coronal and sagittal sutures [1].

The condition has an estimated prevalence of 1 in 25,000 live births worldwide [2]. CS constitutes approximately 4%–8% of all craniosynostosis cases. It is caused by a mutation in the fibroblast growth factor receptor‐2 (FGFR2) gene [3]. Presentation usually occurs in early childhood due to characteristic craniofacial features [4]. Although recurrent upper respiratory tract infections and obstructive sleep apnoea are recognized manifestations of the syndrome, severe pansinusitis with orbital and neurological complications as the presenting feature is extremely rare.

We report a previously undiagnosed adolescent patient with Crouzon syndrome who presented with pansinusitis, orbital cellulitis, frontal bone osteomyelitis, and altered mentation requiring urgent surgical drainage. This patient was successfully managed with FESS and antimicrobial therapy, which constitutes a unique combination of interventions in patients with Crouzon′s syndrome.

## 2. Case Presentation

A 14‐year‐old boy presented with fever, headache, bilateral progressive periorbital swelling, left eye discharge, and worsening drowsiness of 7 days′ duration. The illness began with high‐grade fever and headache, followed by bilateral eyelid swelling and mucopurulent discharge from the left eye. Subsequently, he developed left‐sided proptosis, restricted ocular movements, lethargy, confusion, and altered sensorium. Greenish nasal discharge appeared on the fourth day of illness. He was initially managed at a local hospital for orbital cellulitis and meningitis but was referred to our tertiary care center due to progressive clinical deterioration.

The patient had a history of recurrent upper respiratory tract infections. There was no prior diagnosis of craniosynostosis. Past history did not reveal any records of eye disease or prior maxillofacial surgeries. There was no known family history of ophthalmic disease, craniofacial anomalies, or consanguinity.

**Figure 1:**
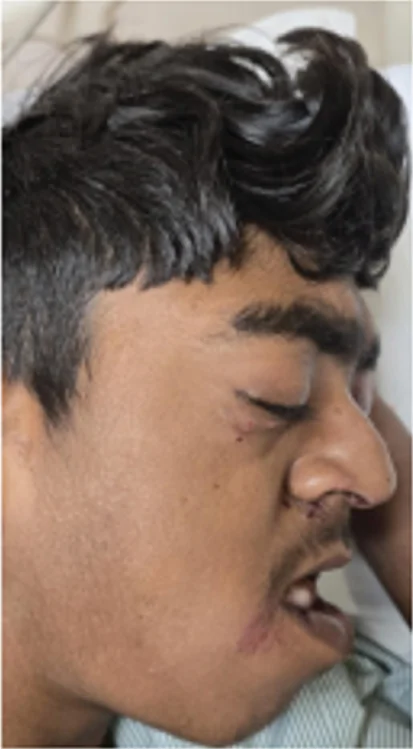
Characteristic craniofacial abnormalities included midfacial hypoplasia, shallow orbits, mandibular prognathism, and Grade III malocclusion.

**Figure 2:**
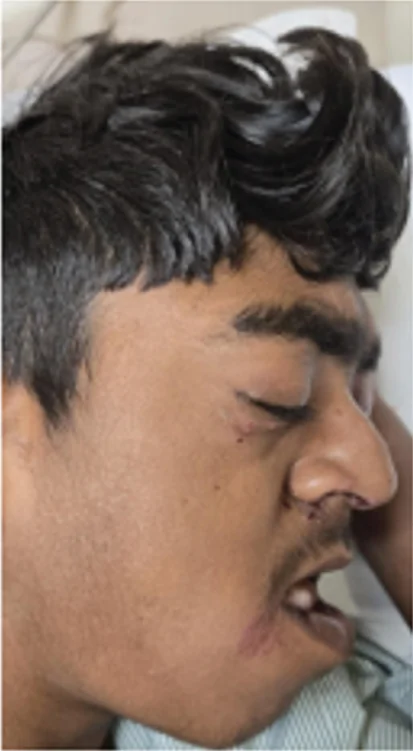
The image shows elongated face, parrot‐beak nasal deformity, and hypertelorism with bilateral proptosis. The left eye showed excoriation, purulent discharge, and conjunctival chemosis.

On examination, he was drowsy and disoriented. Characteristic craniofacial abnormalities included midfacial hypoplasia, shallow orbits, bilateral proptosis, mandibular prognathism, and Grade III malocclusion. The left eye showed conjunctival chemosis, purulent discharge, and diminished vision. Bilateral mucopurulent nasal discharge was present (Figures 1 and 2). Neurological examination was limited because of altered sensorium. Other parts of the skeletal system were unremarkable.

A provisional diagnosis of orbital cellulitis ± meningitis was made, and empirical antimicrobial therapy was initiated with injection vancomycin, ceftriaxone, and metronidazole.

Lab investigations showed elevated total leucocyte count (25,000/mm^3^) with neutrophilic predominance (89%). C‐reactive protein (114 mg/L) and procalcitonin (5.7 ng/mL) levels were also elevated. Pus swab from the nasal and ocular discharge along with the blood samples was sent for microbiological cultures.

**Figure 3:**
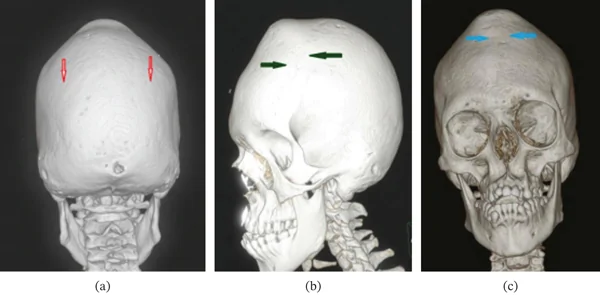
Computed tomography with three‐dimensional reconstruction of the skull showing elongated tower‐shaped skull (oxycephaly) with prematurely fused lambdoid (a, red arrows), coronal (b, green arrows), and sagittal (c, blue arrows) sutures due to craniosynostosis. All the three sutures are nonvisualized.

**Figure 4:**
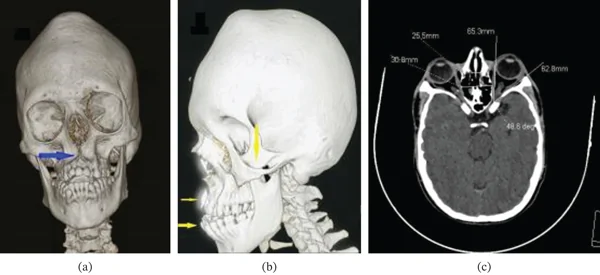
A 3D reconstruction of skull showing high‐arched cleft palate (a, blue arrow). (b) Sagittal view showed mandibular prognathism with maxillary and zygomatic hypoplasia (yellow arrows). (c) Shallow orbits with hypertelorism was diagnosed in axial view. The anterior orbital and midorbital distance were 25.5 and 30.84 mm, respectively, with a lateral wall angle of 48.6°, which was diagnostic of hypertelorism and shallow orbits.

Computed tomography with three‐dimensional reconstruction showed an abnormal shape of the skull (oxycephaly) due to craniosynostosis. Coronal, sagittal, and lambdoid sutures were already fused (Figure 3a–c), which was premature for his age. Other findings included high‐arched cleft palate (Figure 4a), mandibular prognathism with maxillary, and zygomatic hypoplasia (Figure 4b). On measuring, the lateral wall angle (48.6° vs. normal < 41.74°), anterior orbital (25.5 vs. normal < 25.9 mm), and midorbital distance (30.84 < 1.84 mm) were found to be increased, suggestive of shallow orbits and hypertelorism (Figure 4c).

**Figure 5:**
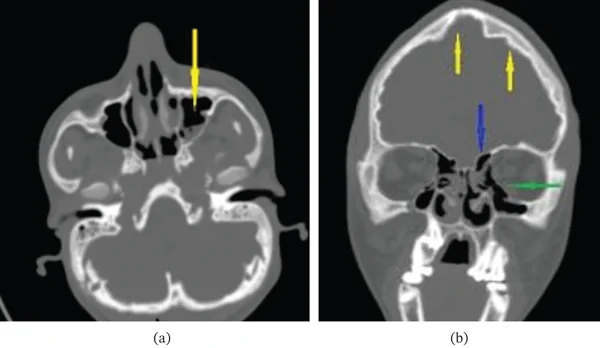
CT paranasal sinuses showing maxillary (a, yellow arrows), sphenoid, frontal and ethmoid sinusitis (b, blue and green arrows). Variable thickness of inner and outer tables with diffuse indentation giving a beaten‐copper appearance to the skull (b, yellow arrows).

CT scan of paranasal sinuses revealed extensive bilateral pansinusitis with left orbital involvement (Figure 5a). Coronal view revealed a narrow/high‐arched palate. Variable thickness of inner and outer tables with diffuse indentation of inner table of the skull was seen, giving a typical beaten‐copper appearance with oxycephaly (tower‐shaped skull) (Figure 5b).

**Figure 6:**
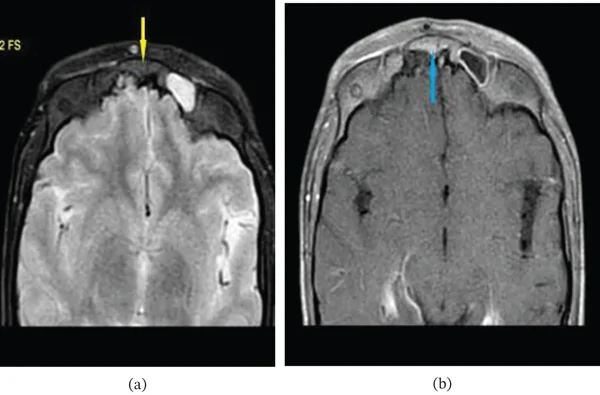
MRI brain with orbits showed T2 Fat Sat hyperintensity (a, yellow arrow) with postcontrast enhancement (b, blue arrow) in the frontal bone with surrounding scalp stranding suggesting osteomyelitis.

MRI T2 Fat Sat images showed hyperintensity of the frontal bone (Figure 6a) and postcontrast enhancement with surrounding scalp tissues (Figure 6b). It confirmed frontal sinusitis, osteomyelitis, and soft tissue involvement. A final diagnosis of pansinusitis mimicking orbital cellulitis with a previously undiagnosed Crouzon′s syndrome was made.

Microbiological cultures subsequently isolated carbapenem‐resistant *Pseudomonas aeruginosa*. Antimicrobial therapy was escalated to intravenous colistin and tigecycline according to susceptibility testing. By the end of Day 3, the patient showed only slight improvement in sensorium.

Following multidisciplinary discussion involving otorhinolaryngology, ophthalmology, radiology, and critical care teams, urgent functional endoscopic sinus surgery (FESS) was undertaken because of progressive orbital cellulitis, frontal osteomyelitis, altered sensorium, and failure of initial medical management. Endoscopic examination demonstrated extensive mucosal edema and purulent secretions involving multiple sinonasal cavities. Particular care was required because of the distorted craniofacial anatomy associated with Crouzon syndrome, including maxillary hypoplasia, shallow orbits, and altered sinonasal landmarks. A conservative endoscopic approach was adopted throughout the procedure. Thick purulent material was evacuated and sent for microbiological analysis.

**Figure 7:**
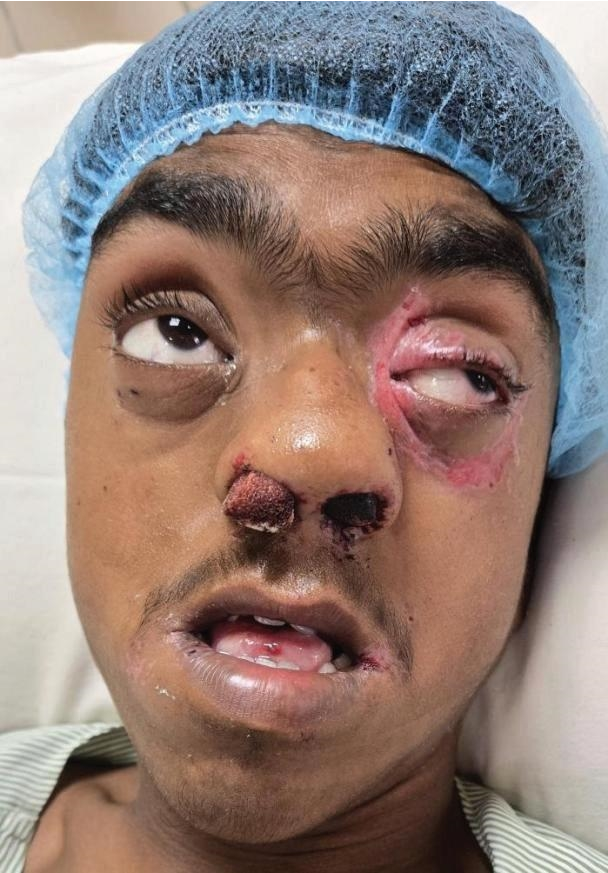
Postoperative Day 2. Significant resolution of eyelid swelling. No active discharge from the nasal cavity or the eye. Improved sensorium and alertness.

**Figure 8:**
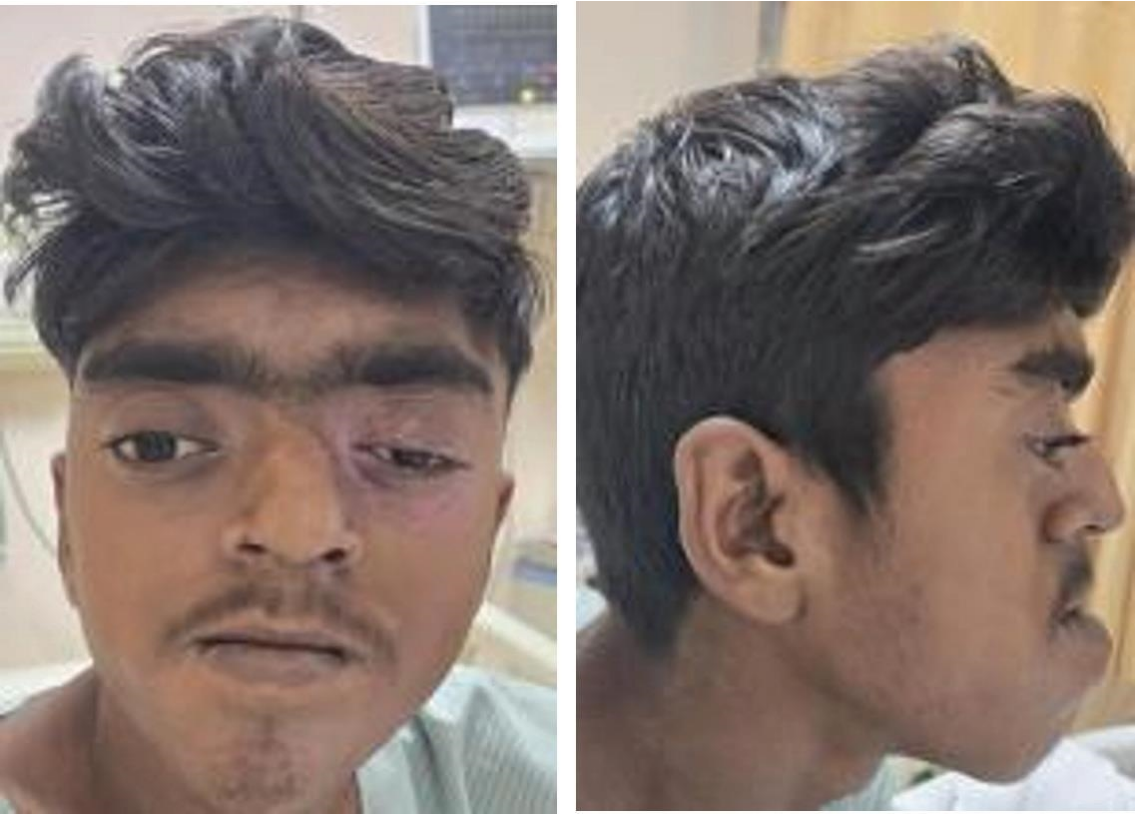
At the time of discharge, after complete resolution of nasal and ocular symptoms. Hypertelorism, proptosis, elongated face with midfacial hypoplasia, prognathism, malocclusion, and parrot beak deformity are more evident.

The patient showed marked clinical improvement within 48 h. Orbital swelling reduced significantly, sensorium normalized, and visual symptoms improved without requiring orbital decompression (Figure 7). Minimally invasive surgery helped in faster recovery and shorter length of stay. Patient was discharged home after a satisfactory recovery (Figure 8). At a 3‐month follow‐up, the patient was free of any symptoms related to sinusitis. Subsequent genetic testing confirmed an FGFR2 mutation consistent with Crouzon syndrome.

## 3. Discussion

CS results in premature fusion of cranial sutures leading to the classical triad of brachycephaly, midfacial hypoplasia, and proptosis [5]. Previously undiagnosed Crouzon syndrome presenting during adolescence usually has a milder phenotypic expression. Complete phenotypes rarely present with severe infective complications such as pansinusitis, orbital cellulitis, frontal osteomyelitis, and altered mentation in their late adolescence. Delayed diagnosis in our patient might be a consequence of limited access to specialized craniofacial care [6]. The present case is noteworthy because diagnosis was established in adolescence only after the development of severe infective complications.

Patients with Crouzon syndrome are predisposed to sinonasal disease because of midfacial hypoplasia, abnormal skull base development, narrowing of sinonasal drainage pathways, and altered mucociliary clearance. These factors may contribute to recurrent rhinosinusitis with increased risk of orbital or intracranial spread of infection. Although there is no singular established statistical incidence rate for craniosynostosis causing rhinosinusitis, these structural craniofacial abnormalities frequently result in chronic rhinosinusitis [7]. Despite a strong association between recurrent sinonasal infections and syndromic craniosynostosis, severe pansinusitis complicated by orbital cellulitis, frontal bone osteomyelitis, and altered mentation is rarely reported as the presenting manifestation of Crouzon syndrome [6].

Management of sinusitis in CS follows the general principles applied to chronic rhinosinusitis, with medical therapy as the first line of management. However, the underlying anatomic obstruction may limit therapeutic success. Surgical intervention becomes necessary when there is orbital involvement, intracranial extension, osteomyelitis, neurological deterioration, or failure of conservative treatment.

FESS in patients with CS presents unique challenges because of distorted anatomy, altered skull‐base orientation, shallow orbits, maxillary hypoplasia, and lamina papyracea abnormalities. Careful preoperative radiological evaluation and meticulous surgical technique are therefore essential to reduce the risk of complications. In a case series of seven syndromic patients, Bradford A. et al. [7] stated that in critical scenarios, endoscopic sinus surgery can be safely and effectively performed in patients with craniofacial malformations. They observed abnormal sinus anatomy in all patients, most commonly, skull base abnormalities and dehiscent lamina papyracea. In the present case, timely culture‐directed antimicrobial therapy and urgent surgical decompression with FESS resulted in rapid clinical improvement and complete recovery. The successful outcome in this case highlights that FESS, when performed judiciously, can provide significant symptomatic relief even in syndromic craniosynostosis patients [8]. This outcome underscores the importance of multidisciplinary management involving otorhinolaryngologists, ophthalmologists, radiologists, and intensivists.

## 4. Conclusion

Undiagnosed CS may present late in adolescence or even early adulthood with atypical and severe life‐threatening complications such as pansinusitis and orbital cellulitis. It is imperative to have awareness of the syndromes associated with craniofacial dysmorphism for correct identification and appropriate management of such patients. Although challenging, FESS can be safely and effectively performed in craniosynostosis patients with careful preoperative assessment and a conservative surgical philosophy. Reporting such cases adds to the limited literature on managing sinonasal diseases in patients with craniosynostosis.

**Nomenclature**
CS: Crouzon′s syndrome
CT: computerized tomography
FESS: functional endoscopic sinus surgery
MRI: magnetic resonance imaging

## Author Contributions

P.I. and S.G. conceptualized the article, drafted the manuscript, and reviewed the literature. R.G. did the research and made a radiological diagnosis. A.B. reviewed the script.

## Funding

No funding was received for this manuscript.

## Disclosure

All authors have read and approved the final version of the manuscript. Dr. Parul Issar, as manuscript guarantor, had full access to all clinical data included in this report and takes complete responsibility for the integrity of the data and the accuracy of the information presented.

## Ethics Statement

Our institution does not require ethical approval for reporting individual cases.

## Consent

A written informed consent was obtained from the patient′s family for the publication of this case study.

## Conflicts of Interest

The authors declare no conflicts of interest.

## Data Availability

The data that support the findings of this study are available on request from the corresponding author. The data are not publicly available due to privacy or ethical restrictions.
